# Identification and Genome Characterization of a Novel Nege-like Virus Isolated from Aphids (*Aphis gossypii*) in Yunnan Province

**DOI:** 10.3390/ijms25115802

**Published:** 2024-05-26

**Authors:** Mengying Hua, Linhui Song, Jiaying Wang, Jian Wu, Jianfeng Gu, Suwen Huang, Weijun Duan, Fei Yan, Zhongtian Xu, Jiejun Peng

**Affiliations:** 1 State Key Laboratory for Managing Biotic and Chemical Threats to the Quality and Safety of Agroproducts, Institute of Plant Virology, Ningbo University, Ningbo 315211, China; huamengying04@163.com (M.H.); songlinhui0622@163.com (L.S.); wujian@nbu.edu.cn (J.W.); yanfei@nbu.edu.cn (F.Y.); 2 Key Laboratory of Biotechnology in Plant Protection of MARA and Zhejiang Province, Institute of Plant Virology, Ningbo University, Ningbo 315211, China; 3 Ningbo Key Laboratory of Port Biological and Food Safety Testing, Ningbo Customs Technology Center (Ningbo Inspection and Quarantine Science Technology Academy), 8 Huikang, Ningbo 315100, China; wangjiaying@customs.gov.cn (J.W.); jeffgu00@163.com (J.G.); hsw@nbyjg.com (S.H.); weijunduan@163.com (W.D.)

**Keywords:** negevirus, nege-like virus, *Aphis gossypii*, AlphaFold2, high-throughput transcriptome sequencing

## Abstract

Negeviruses are insect-specific enveloped RNA viruses that exhibit a wide geographic distribution. A novel nege-like virus, tentatively named *Aphis gossypii* nege-like virus (AGNLV, GenBank: OR880429.1), was isolated from aphids (*Aphis gossypii*) in Lijiang City, Yunnan, China. AGNLV has a genome sequence of 9258 nt (excluding the polyA tail) encoding three open reading frames (ORFs). ORF1 (7149 nt) encodes a viral methyltransferase, a viral RNA helicase, and an RNA-dependent RNA polymerase. ORF2 (1422 nt) encodes a DiSB-ORF2_chro domain and ORF3 encodes an SP24 domain. The genome sequence of AGNLV shares the highest nucleotide identity of 60.0% and 59.5% with Wuhan house centipede virus 1 (WHCV1) and Astegopteryx formosana nege-like virus (AFNLV), respectively. Phylogenetic analysis based on the RNA-dependent RNA polymerase shows that AGNLV is clustered with other negeviruses and nege-like viruses discovered in aphids, forming a distinct “unclassified clade”. Interestingly, AGNLV only encodes three ORFs, whereas AFNLV and WHCV1 have four ORFs. Structure and transmembrane domain predictions show the presence of eight alpha helices and five transmembrane helices in the AGNLV ORF3. Translational enhancement of the AGNLV 5′ UTR was similar to that of the 5′ UTR of plant viruses. Our findings provide evidence of the diversity and structure of nege-like viruses and are the first record of such a virus from a member of the genus *Aphis*.

## 1. Introduction

Negeviruses are a currently unclassified group of insect-specific viruses with a positive-sense, single-stranded RNA genome of 9 to 10 kb. They have been reported from America, Europe, Africa, and Asia and belong to two main clades, for which the names Nelorpivirus and Sandewavirus have been suggested [1,2,3,4,5,6,7,8]. The lengths of their 5′ and 3′ untranslated regions (UTRs) vary significantly, with the 5′ UTR ranging from 72 to 730 nucleotides, and the 3′ UTR spanning 121 to 442 nucleotides with a poly A tail of 13 to 52 nucleotides [7]. It is uncertain whether they have a typical 5′ cap or a virus genome-linked protein (VPg), but negeviruses can be rescued in vitro without the use of a cap analogue for RNA synthesis. However, this process is delayed when compared to their capped RNA counterparts, suggesting that negeviruses may utilize a cap-independent mechanism for RNA synthesis, and this merits further investigation to fully understand their replication strategies. The secondary structure of the 5′ UTR of negeviruses has been predicted to contain an internal ribosome entry site (IRES) at its 5′ end [9]. This suggests that negeviruses may employ a cap-independent mechanism for initiating translation, which is crucial for understanding their replication and protein synthesis strategies. Most negeviruses have three primary open reading frames (ORFs) in their genomes. ORF1 encodes the RNA-dependent RNA polymerase (RdRp) while ORFs 2 and 3 encode glycoproteins and membrane proteins, respectively [5,7]. An additional protein (ORF4), which lacks conserved domains, is present in a few negeviruses [10,11,12]. This variation in genomic structure highlights the evolutionary diversity and potential adaptability of these viruses.

Structural studies on negeviruses have highlighted a novel virion structure primarily composed of the two structural proteins encoded by ORF2 and ORF3. Glycoproteins form short projections, while the membrane proteins form an elliptical core [13]. As in plant viruses, the virions change shape in response to pH variations. In acidic environments, negevirus cores transform into bullet-like or tubular structures. The short projections on the virion may be crucial for facilitating entry into insect host cells [13].

Aphids, members of the order Hemiptera in the family *Aphididae*, are prominent pests with a considerable impact on agricultural and horticultural crops. They are known to host numerous novel insect-specific viruses (ISVs) from families such as *Dicistroviridae* and *Iflaviridae*, in addition to members of unclassified groups [10,14]. Recently, several nege-like viruses have been reported in aphids [10,15,16], and this study presents a comprehensive characterization of a novel nege-like virus derived from the cotton aphid (*Aphis gossypii*).

## 2. Results and Discussion

In total, 20 aphids were collected from the leaves of *Vicia faba* L. in Lijiang, Yunnan Province, China. To investigate the virus(es) present, total RNA was extracted, purified and subjected to high throughput RNA-sequencing. A total of 22,175,795 paired-end reads was acquired, yielding 44,979 de novo contigs. These contigs were subsequently compared to NCBI reference viruses using BLASTx, and 5 contigs were identified with E-values of zero. Specifically, Cluster-24828.2658_1, spanning 9250 nucleotides, exhibited a 39.7% amino acid identity to the RdRP of *Astegopteryx formosana* nege-like virus (AFNLV). To ascertain the aphid species, all 44,979 contigs were cross-referenced with the cytochrome oxidase subunit 1 (COI) database of Barcode of Life Data Systems Version 4 (http://www.boldsystems.org (accessed on 12 November 2023) using BLASTn. The outcomes strongly indicated that the aphid species was *A. gossypii*, with a robust 99.6% sequence identity with a COI sequence from a conspecific aphid, cataloged under accession no. MN083248.1 in the NCBI database. Consequently, the provisionally designated novel virus was named *Aphis gossypii* nege-like virus (AGNLV).

**Figure 1:**
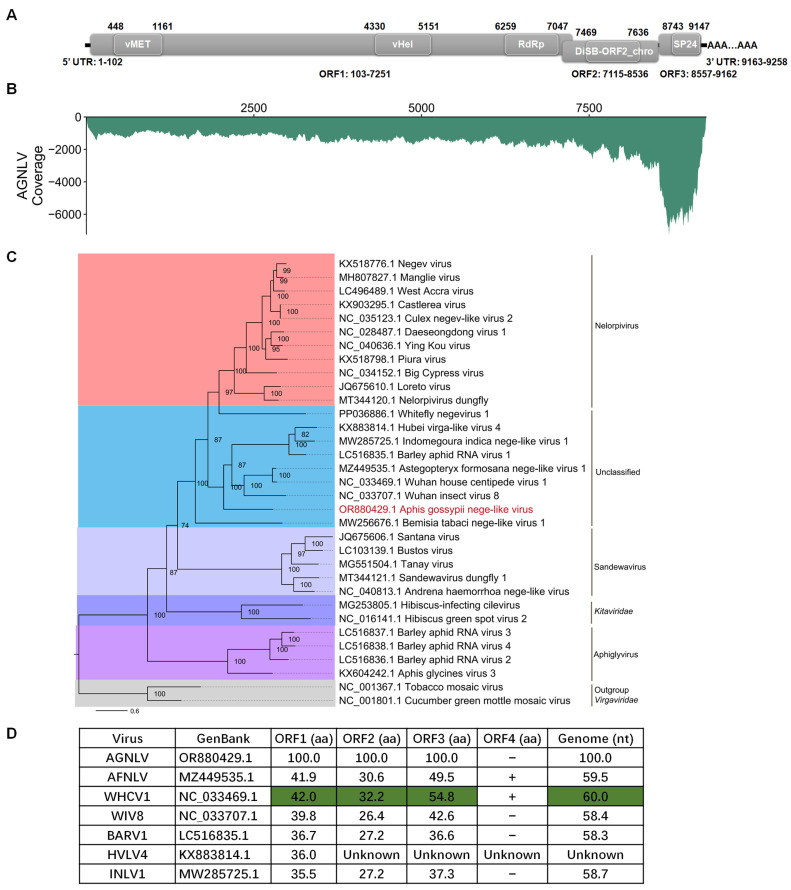
(**A**,**B**) Genome structure and transcriptome raw read coverage of Aphis gossypii nege-like virus (AGNLV). vMET, a viral methyltransferase; vHel, a viral RNA helicase; RdRp, an RNA-dependent RNA polymerase; UTR, untranslated region. (**C**) Maximum likelihood phylogenetic tree based on AGNLV and related viruses with tobacco mosaic virus (TMV) and cucumber green mottle mosaic virus (CGMMV) as outgroup. (**D**) ORF amino acid genome nucleotide comparisons between AGNLV and related viruses. Green shading is used for the highest identities.

To obtain the full-length sequence of AGNLV, the 3′ RACE, 5′ RACE, and three overlapping sequence fragments were generated using the primer pairs listed in Table 1. The complete genome RNA of AGNLV (GenBank: OR880429.1) has 9258 nucleotides (excluding the polyA tail) and has three predicted open reading frames (ORFs) (Figure 1A). Domain prediction was conducted using CD-Search (https://www.ncbi.nlm.nih.gov/Structure/cdd/wrpsb.cgi (accessed on 12 November 2023). ORF1 was found to comprise three domains, namely a viral methyltransferase (vMet, aa 116-353; Accession, cl03298), a viral RNA helicase (vHel, aa 1410-1683; Accession, pfam01443), and a catalytic core domain of RNA-dependent RNA polymerase in the family *Kitaviridae* (RdRp, aa 2053-2315; Accession, cd23254). This configuration is consistent with other negeviruses [4,7,8,10]. ORF2 and ORF3 encode proteins featuring putative DiSB-ORF2_chro (aa 119-174; Accession, cl24918) and SP24 domains (aa63-197; Accession, pfam16504), respectively. To assess the abundance of AGNLV, clean reads from the transcriptome were aligned back to the full genome sequence of AGNLV using Bowtie2 [17]. Subsequently, the coverage of AGNLV was calculated using Samtools [18]. Notably, viral reads were concentrated within the 3′ terminus of the genome, particularly in ORF3 (Figure 1B). This observation suggested the possible presence of viral sub-genomic RNAs in this region.

The amino acid sequences of AGNLV ORF1 showed identities of 38.8% and 39.0% with *Astegopteryx formosana* nege-like virus (AFNLV, MZ449535) and Wuhan house centipede virus 1 (WHCV1, NC_033469.1) using BLASTp. To unravel the evolutionary path of AGNLV, the RdRp amino acid sequences of AGNLV and other nege/nege-like viruses were used to construct a phylogenetic tree [10,15,16,19]. Evolutionary analyses showed that Wuhan insect virus 8 (WIV8, NC_033707.1), AGNLV, and WHCV1 clustered into one subgroup, while Barley aphid RNA virus 1 (BARV1, LC516835.1), Hubei virga-like virus 4 (HVLV4, KX883814.1), and Indomegoura nege-like virus 1 (INLV1, MW285725.1) clustered into another subgroup (Figure 1C). AGNLV was similar to WIV8, AGNVL, WHCV1, BARV1, HVLV4 and INLV1 (Figure 1C,D). The alignment of amino acid sequences from AGNLV proteins with those of related viruses indicated that the ORFs of AGNLV exhibited the highest identities with AFNLV and WHCV1. Furthermore, the alignment of genome sequences also highlighted that AGNLV shared the highest identities with AFNLV and WHCV1 (Figure 1D). Although AFNLV and WHCV1 both encoded four ORFs (RdRp, virion glycoprotein, virion membrane protein, and a hypothetical protein), AGNLV encoded only the first three ORFs. Recent research shows that nege-like viruses have been reported in agriculturally important plant-feeding arthropods, such as aphids and whiteflies [10,15,16,19]. Through in-depth genome comparisons and phylogenetic analyses, we uncovered the evolutionary associations of this virus with other negeviruses that have the capability to infect both arthropods and plants.

**Figure 2:**
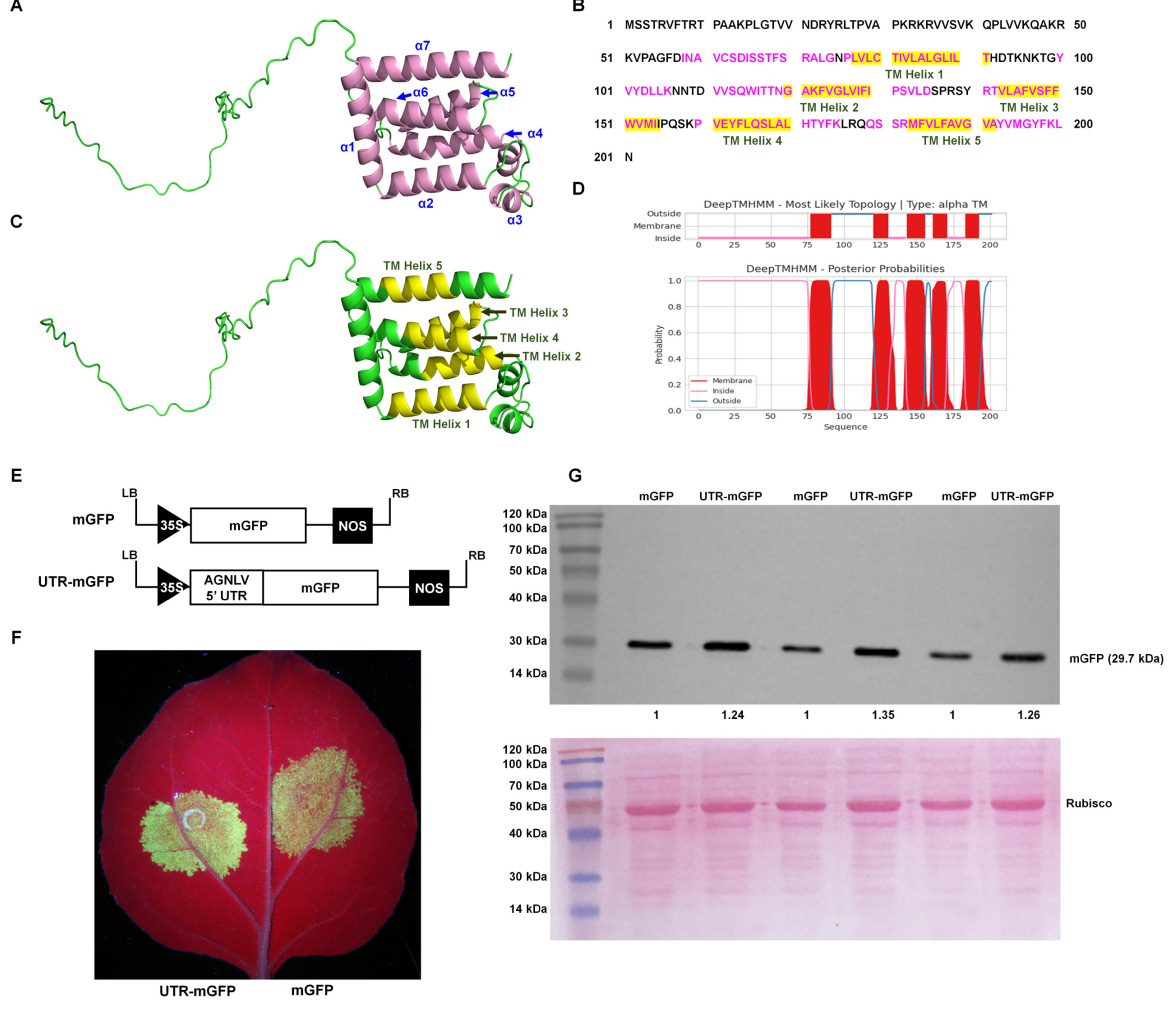
Predictive analysis of AGNLV major envelope protein and 5′ UTR structures. (**A**,**C**) Predictions by AlphaFold2 [20] and PSIPRED [21], illustrate the ORF3 protein structure with seven alpha helices(indicated in pink). (**B**–**D**) Analysis of transmembrane helices within the AGNLV ORF3 major envelope protein using the DeepTMHMM Server [23] and AlphaFold2, with five helices depicted in yellow. (**E**) Schematic representation of 5′ UTR-mGFP and mGFP transient expression vectors. (**F**) Visualization of *N. benthamiana* plants inoculated with UTR-mGFP and mGFP vectors under UV light at 2 dpi. (**G**) Western blot analysis confirming the accumulation of GFP proteins in inoculated leaves of *N. benthamiana* plants.

AlphaFold, an advanced deep learning algorithm, significantly enhanced the accuracy of protein structure prediction. This computational tool facilitated the detailed prediction and analysis of protein structures, potentially streamlining viral research processes. The major envelope protein of negeviruses encoded by ORF3 played a pivotal role in the maturation of virions, indicating its critical importance in the viral life cycle. The AlphaFold2 algorithm (UCSF ChimeraX version 1.70) and the PSIPRED tool [20,21] predicted that the AGNLV ORF3 protein has two segments: a disordered region from amino acids 1 to 57, and a region of alpha helices from amino acids 58 to 201 (Figure 2A,C and File S1 and S2). There was a complex arrangement of alpha helices, with eight identified by AlphaFold2 and seven by PSIPRED (File S1). Research on Negeviruses, particularly the Tanay virus (TANVA), mostly used cryo-electron microscopy (cryo-EM) single-particle analysis (SPA) and cryo-electron tomography (cryo-ET) to determine their 3D virion structures under nearly native conditions, but the crystal structure of ORF3 remains elusive [13]. Predicting protein three-dimensional structures was inherently challenging. AlphaFold2, through its deep learning approach, predicted protein distances and torsion angles with high precision, utilizing training data from experimentally verified PDB structures, primary protein sequences, and multiple sequence alignments (MSAs) [22]. The notable differences observed between the predictions from AlphaFold2 and PSIPRED for ORF3 could be primarily attributed to the non-availability of reference structural data for this protein. The transmembrane domains of AGNLV ORF3 were predicted using the DeepTMHMM Server (https://dtu.biolib.com/DeepTMHMM (accessed on 9 March 2024)) [23]. Altogether, the application of structural and conserved domain prediction tools had facilitated the delineation of a hypothetical structure and function for viral proteins, potentially advancing research into their evolutionary and functional studies.

The Internal Ribosome Entry Site (IRES) was a crucial cis-acting RNA element in human, animal, and plant plus-strand RNA viruses [24,25]. In vitro transcription experiments with Negev virus (NEGV) RNA demonstrated that the virus could be rescued without a cap analogue. This finding supported the hypothesis that an IRES at the 5′ end of the negevirus genome mediated cap-independent translation, and provided a mechanism for the initiation of protein synthesis under cap-independent conditions [9]. It was well documented that 5′ UTRs of plant viruses contain elements that enhance translational efficiency, characteristic of IRES activity [26,27,28]. To investigate the AGNLV 5′ UTR’s potential translational enhancement, constructed containing a GFP reporter linked to the AGNLV 5′ UTR and GFP alone as a control, were compared in *N. benthamiana* plants. (Figure 2E). Two transient expression vectors were transformed into *A. tumefaciens* and then delivered to *N. benthamiana* plants by infiltration (Leaf abaxial, left: mGFP, right: UTR-mGFP) (Figure 2F). At two days post-inoculation (dpi), UV lamp examination revealed that the GFP fluorescence in plants expressing UTR-mGFP was significantly higher than in those expressing mGFP alone. Western blot analysis confirmed a 1.24~1.35-fold increase in GFP protein levels in UTR-mGFP samples compared to controls (Figure 2G), confirming the role of AGNLV 5′ UTR in enhancing translation, similar to the function of IRES elements in plant viruses.

In summary, AGNLV is a novel nege-like virus isolated from *A. gossypii*, the first such virus discovered from the genus *Aphis*. Its distinctive genomic features, particularly the difference in the number of encoded ORFs, make it an intriguing virus for further studies. Structural predictions and the experimental validation of Internal Ribosome Entry Site (IRES) elements provide a deeper insight into the genetic and functional organization of negeviruses, thereby advancing our knowledge of their evolutionary patterns and molecular biology.

## 3. Materials and Methods

### 3.1. Sample Collection and Total RNA Extration

In April 2023, aphids were collected from the leaves of *Vicia faba* L. in Lijiang, Yunnan Province, China. After freezing in liquid nitrogen to preserve the RNA integrity, total RNA was extracted from a pool of 20 aphids using TRIzol™ Reagent (Invitrogen, Carlsbad, CA, USA), following the manufacturer’s instructions.

### 3.2. RNA Sequencing and De Novo Transcriptome Assembly

To purify mRNA from total RNA, poly-T oligo-attached magnetic beads were utilized. The purified RNA was then fragmented using divalent cations at elevated temperatures with NEB Next First Strand Synthesis Reaction Buffer (5×) (New England Biolabs, Ipswich, MA, USA). Random hexamer primers and MuLV Reverse Transcriptase (RNase H) (New England Biolabs, Ipswich, MA, USA) facilitated the synthesis of the first strand of cDNA. The second strand was synthesized using RNase H and DNA Polymerase I (New England Biolabs, Ipswich, MA, USA). Subsequently, the 3′ ends of DNA fragments were adenylated, and the NEB Next Adaptor with a hairpin loop structure was ligated to prepare the fragments for hybridization. The library fragments underwent purification with the AMPure XP system (Beckman Coulter, Beverly, USA) to select cDNA fragments between 370–420 bp in length. After size selection, the adaptor-ligated cDNA was incubated at 37 °C for 15 min, followed by 5 min at 95 °C to prepare for PCR. PCR amplification was performed using Index (X) Primer, Universal PCR primers and Phusion High-Fidelity DNA Polymerase (New England Biolabs, Ipswich, MA, USA). The PCR products were finally purified using the AMPure XP system, then the library quality was assessed on an Agilent Bioanalyzer 2100 (Agilent Technologies, Santa Clara, CA, USA).

Transcriptome sequencing was conducted on the Illumina NovaSeq 6000 platform (Illumina, San Diego, CA, USA) using 150 bp paired-end reads. Data analysis was performed with CLC Genomics Workbench 20 (QIAGEN, Duesseldorf, Germany). For each library/sample, the left reads (read1 files) and the right reads (read2 files) were combined into two separate files, named left.fq and right.fq, respectively. Transcriptome assembly was carried out using Trinity (Trinityrnaseq-v2.15.1, Broad Institute) with the minimum k-mer coverage parameter (min_kmer_cov) set to 2, and all other parameters remained at their default settings.

### 3.3. Viral Contig Identification

To identify and annotate virus-associated contigs, the assembled transcriptome contigs were analyzed using the BLASTx tool against the viral sequence database available at the National Center for Biotechnology Information (NCBI) (https://www.ncbi.nlm.nih.gov (accessed on 25 October 2023). Subsequently, clean reads were aligned to the identified viral contigs using the Burrows–Wheeler Aligner (BWA) program with default parameters [29].

### 3.4. RACE and Overlapping RT-PCR

To determine the full-length sequence of the candidate virus, the 3′-end first strand cDNA was synthesized using M4T primers and the ReverTra Ace™ qPCR RT Kit (TOYOBO, Osaka, Japan) following the manufacturer’s protocol (Table 1).

PCR amplification was performed using M4 and 3′ RACE-AGNLV-1 F primers with KOD-plus-Neo (TOYOBO, Osaka, Japan) [30]. RT-PCR reactions were conducted in a 50-μL mixture comprising 1.0 μL of cDNA, 1.0 μL of each forward and reverse primer (10 μM), 25 μL of 2× PCR buffer for KOD, 10 μL of 2 mM dNTPs (TOYOBO, Osaka, Japan), and 12 μL of water across six tubes. The thermal cycling conditions were set as follows: an initial denaturation at 98 °C for 5 min, followed by 35 cycles of 98 °C for 30 s, annealing between 50–70 °C for 30 s, extension at 68 °C for 1 min, and a final extension at 68 °C for 10 min. The first-round PCR products were pooled and diluted 100-fold for subsequent amplification. The second-round PCR used M4 and 3′ RACE-AGNLV-2 F primers, as described above, followed by the cloning and sequencing of the candidate segments. 

For 5′ RACE, first strand cDNA synthesis utilized a gene-specific reverse primer (5′ RACE-AGNLV-1 R) and an adapter primer (ZHM1) which were ligated to the cDNA/RNA duplexes using T4 RNA ligase (TaKaRa, Dalian, China) [31]. The first-round PCR product of 5′ RACE was amplified using ZHM2 and 5′ RACE-AGNLV-1 R primers, and the second-round PCR used ZHM2 and 5′ RACE-AGNLV-2 R, with the subsequent cloning and sequencing of the segments. 

To verify the integrity and authenticity of the full-length sequences of the candidate virus, three overlapping sequence fragments covering the entire genome were amplified using primer pairs AGNLV-1, AGNLV-2, and AGNLV-3 (Table 1).

### 3.5. Construction of Phylogenetic Trees

The amino acid sequences of the newly identified virus RNA-dependent RNA polymerase (RdRP), along with representative sequences from the NCBI nucleotide database, were aligned using MAFFT (version 7.0) [10,32]. Poorly aligned regions and spurious sequences were removed with TrimAl [33]. Phylogenetic analysis was conducted using IQ-TREE (v1.6.6) [34], employing the maximum likelihood method with the best-fit amino acid substitution model identified by ModelFinder [35]. The confidence of the tree was assessed through 5000 ultrafast bootstrap replicates.

### 3.6. Stucture and Transmembrane Domain Predictions

Protein structures were predicted using the AlphaFold2 algorithm within UCSF ChimeraX (v1.7). UCSF ChimeraX runs AlphaFold on Google Colab free servers [20]. Secondary structure predictions, disorder, membrane helix and profile-based fold recognition of viral proteins were carried out using the PSIPRED Workbench (http://bioinf.cs.ucl.ac.uk/psipred/ (accessed on 9 March2024) [21]. Transmembrane domain predictions were carried out using the DeepTMHMM server (https://dtu.biolib.com/DeepTMHMM (accessed on 11 March 2024).

### 3.7. Plant Agroinfiltration and Western Blot

*Nicotiana benthamiana* plants were cultured in a light incubator with 14 h of light and 8 h of darkness at 26 °C. To confirm the enhancer function of AGNLV 5′ UTR, the plasmids (mGFP or 5′ UTR-mGFP) were transformed into *Agrobacterium tumefaciens* (C58C1) (Weidibio, Shanghai, China) which was then delivered to *Nicotiana benthamiana*.

Total proteins from *N. benthamiana* leaf samples (1 cm diameter) were extracted using a protein extraction buffer composed of 50 mM sodium phosphate buffer (pH 7.0), 5 mM β-mercaptoethanol, 10 mM EDTA, and 0.1% Triton X-100 (Sigma-Aldrich, St. Louis, MO, USA). The extracted proteins were mixed with 5× loading buffer and separated by 12% SDS–polyacrylamide gel electrophoresis (PAGE). Protein detection was performed as previously reported [36].

## Figures and Tables

**Table 1:** Primers used in this study.

Target	Name	Sequence (5′-3′)
Full-genome sequence AGNLV	AGNLV-1 F	TAACGATATCTCGCTAAGAGGTGTCATTTT
	AGNLV-1 R	CCCTTTGATTCGGTGTACCC
	AGNLV-2 F	ACAAGCAGACCCATAAGTAGTG
	AGNLV-2 R	GCAACGGTCAAACAACGTCT
	AGNLV-3 F	TAGCGGTCGAAAAAAGGAACTT
	AGNLV-3 R	ACCGTCTAATAAAGTCTAATGAAAT
Virus detection	AGNLV-SP24 F	ATGAGTTCTACACGTGTGTTTA
	AGNLV-SP24 R	TTAATTCAATTTAAAATAGCCCATGAC
5′ and 3′ RACE	M4	GTTTTCCCAGTCACGAC
	M4T	GTTTTCCCAGTCACGAC(T)_15_
	ZHM1	PO_4_-CTCTTCCCCTCCCTCCTC-NH_2_
	ZHM2	GAGGAGGGAGGGGAAGAG
	3′ RACE-AGNLV-1 F	TGTACTATTGTTCTCGCACTCG
	3′ RACE-AGNLV-2 F	AGTACTTTTTGCAATCTCTTGC
	5′ RACE-AGNLV-1 R	CTCGTACGACACCTTACGGTG
	5′ RACE-AGNLV-2 R	GCTCGGCCTGGACAAGAACG

## Data Availability

The genome sequence of AGNLV was deposited in the NCBI GenBank database under accession numbers OR880429.1.

## Supplementary Materials

The supporting information can be downloaded at: https://www.mdpi.com/article/10.3390/ijms25115802/s1.
